# NIRS-BIDS: Brain Imaging Data Structure Extended to Near-Infrared Spectroscopy

**DOI:** 10.1038/s41597-024-04136-9

**Published:** 2025-01-27

**Authors:** Robert Luke, Robert Oostenveld, Helena Cockx, Guiomar Niso, Maureen J Shader, Felipe Orihuela-Espina, Hamish Innes-Brown, Stephen Tucker, David Boas, Meryem A. Yücel, Remi Gau, Taylor Salo, Stefan Appelhoff, Christopher J. Markiewicz, David McAlpine, The BIDS Maintainers, Remi Gau, Taylor Salo, Stefan Appelhoff, Christopher J. Markiewicz, Luca Pollonini

**Affiliations:** 1 Macquarie University Hearing & Department of Linguistics, Australian Hearing Hub, Sydney, Australia; 2 AE Studio, Venice, CA USA; 3 Donders Institute for Brain, Cognition and Behaviour, Radboud University, Nijmegen, the Netherlands; 4 NatMEG, Karolinska Institutet, Stockholm, Sweden; 5 Instituto Cajal and Cajal International Neuroscience Center, CSIC, Madrid, Spain, CSIC, Madrid, Spain; 6 Department of Speech, Language, and Hearing Sciences, Purdue University, West Lafayette, IN USA; 7 School of Computer Science, University of Birmingham, Birmingham, United Kingdom; 8 Eriksholm Research Institute, Snekkersten, Denmark; 9 Neurophotonics Center, Department of Biomedical Engineering, Boston University, Boston, MA USA; 10 Institute of Psychology, Université Catholique de Louvain, Louvain la Neuve, Belgium; 11 Lifespan Informatics & Neuroimaging Center, University of Pennsylvania, Philadelphia, PA USA; 12 Center for Adaptive Rationality, Max Planck Institute for Human Development, Berlin, Germany; 13 Department of Psychology, Stanford University, Stanford, CA USA; 14 Department of Engineering Technology, University of Houston, Houston, TX USA; 15 Basque Center on Cognition, Brain and Language, Donostia-San Sebastián, Spain

**Keywords:** Research data, Neuroscience, Scientific data, Software

## Abstract

Functional near-infrared spectroscopy (fNIRS) is an increasingly popular neuroimaging technique that measures cortical hemodynamic activity in a non-invasive and portable fashion. Although the fNIRS community has been successful in disseminating open-source processing tools and a standard file format (SNIRF), reproducible research and sharing of fNIRS data amongst researchers has been hindered by a lack of standards and clarity over how study data should be organized and stored. This problem is not new in neuroimaging, and it became evident years ago with the proliferation of publicly available neuroimaging datasets. To solve this critical issue, the neuroimaging community created the Brain Imaging Data Structure (BIDS) that specifies standards for how datasets should be organized to facilitate sharing and reproducibility of science. Currently, BIDS supports dozens of neuroimaging modalities including MRI, EEG, MEG, PET, and many others. In this paper, we present the extension of BIDS for NIRS data alongside tools that may assist researchers in organizing existing and new data with the goal of promoting public disseminations of fNIRS datasets.

## Introduction

Functional near-infrared spectroscopy (fNIRS) is a neuroimaging modality that uses near-infrared light signals to estimate temporal changes in the concentrations of oxygenated and de-oxygenated hemoglobin in brain tissue^1–4^. Functional NIRS measurements are acquired non-invasively via an array of optodes placed on the scalp, in which source optodes emit near-infrared light that is partly absorbed by hemoglobin perfusing the scalp and cerebral cortex. A proportion of the emitted light is back-scattered towards detector optodes that measure light intensity, thus estimating optical absorption in optical channels formed by each source-detector pairing. Because each light source emits lights at two or more wavelengths, and the absorption spectra for oxy- and de-oxy hemoglobin are slightly different, it is possible to further estimate independent changes in the concentrations of oxy- and deoxy-hemoglobin between each source and detector. When these changes in hemoglobin concentrations are linked to external stimuli or experimental tasks, they can provide insight into how cortical hemodynamics are tied to brain function, similarly to the principle of functional magnetic resonance imaging (fMRI), and it is often referred to as functional NIRS. However, NIRS can also be used independently of a stimulus or task, for example, to study brain physiology by measuring global cerebral oxygenation, or even to study muscle physiology when placed over a muscle^4^. The specification hereby described focuses on functional NIRS, although it maintains the possibility to extend to other NIRS applications. Therefore, the specification is more generally defined as NIRS-BIDS, even if in most current instances it embeds fNIRS-specific datasets.

Functional NIRS presents several benefits over other neuroimaging techniques. In addition to its non-invasive and non-ionizing nature, fNIRS is relatively low cost, portable, and silent in operation. The use of light as the measurement signal allows for its use in the general population, as well as in many clinical populations in which other neuroimaging techniques are contra-indicated. Given its widespread utility, fNIRS has become increasingly popular in recent years^5^, with multiple manufacturers offering fNIRS devices for various commercial and research applications. Currently available fNIRS systems vary by optode layout and headgear design, light delivery methods, number of light wavelengths used, level of portability and wearability, and measurement principle, including continuous-wave, time-domain, and frequency-domain, amongst other characteristics.

Although there are differences between the systems, the type of data acquired is similar, and in principle, it is possible to propose a common strategy for data storage and organization that allows for efficient data sharing and usage. Recently, the fNIRS community reached a consensus on adopting a standard file format, SNIRF, which encompasses a wider range of data and metadata relevant to various NIRS sub-modalities (SNIRF)^6^. However, a standard organization of data files that facilitates the importing, processing, and sharing of data and metadata that uniquely and comprehensively describes an experimental study is still lacking.

The Brain Imaging Data Structure (BIDS) is a community-driven standard for organizing and sharing brain imaging data across different laboratories, and is characterized by a hierarchical folder structure used to aggregate and organize large heterogeneous datasets, as well as specifications for providing informative metadata (see https://bids.neuroimaging.io for the entire BIDS specifications). Originally developed for Magnetic Resonance Imaging (MRI)^7^, BIDS has now been extended to other imaging modalities, including Magnetoencephalography (MEG), Electroencephalography (EEG), intracranial Electroencephalography (iEEG), Positron Emission Tomography, genetics data and microscopy^8–12^.

BIDS addresses common open-science pitfalls by following the FAIR principles of findability, accessibility, interoperability, and reusability^13^. In doing so, the BIDS standard promotes reproducibility of research findings by providing a template from which consistent data analysis pipelines and procedures can be created. BIDS has been adopted or endorsed by professional bodies such as the International Neuroinformatics Coordinating Facility (ICNF, https://www.incf.org/sbp/brain-imaging-data-structure-bids) and it is under consideration by other professional bodies.

Notably, BIDS not only promotes data sharing aimed at making large-scale datasets publicly available, but it also enables and encourages enacting strategies towards more reliable datasets^14^. The issue of reliability and repeatability, especially and the individual level, is of great relevance for fNIRS^4^, and one of the expected major impacts of the hereby proposed NIRS-BIDS extension is the development of novel measures and methods for assessment of reliability and validity tested on widely disseminated datasets, as well as solutions for further improvement of data collection and analysis techniques.

In this work, we describe the extension of BIDS to NIRS that enables researchers to take advantage of existing BIDS tooling and facilitating storage of multimodal experimental data. The NIRS-BIDS extension is outlined alongside available tools and examples of publicly available datasets.

## Results

**Fig. 1:**
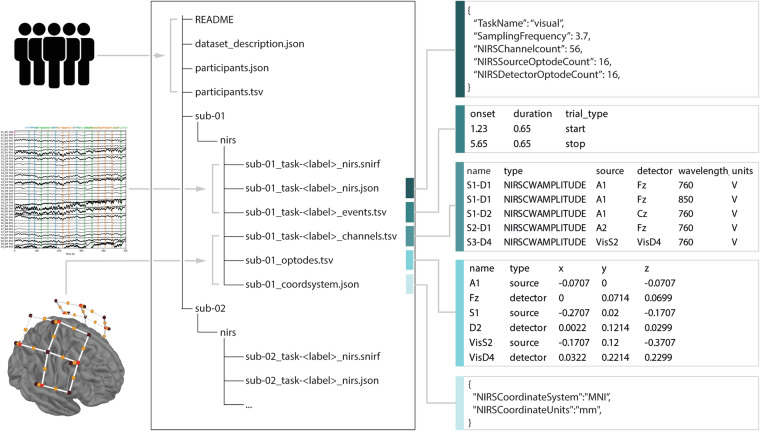
Example of a NIRS-BIDS dataset, including a schematic overview of file content (left), the associated directory structure and files (middle), as well as the specific content of the subject metadata files (right). In this example there is only a single session per participant, hence the optional *session* level is omitted in the directory structure.

The community-driven effort aimed at extending BIDS specifications to include fNIRS data resulted in a hierarchical data structuring that follows modality-agnostic BIDS definitions, yet it describes a new data type *‘nirs’* alongside its relevant metadata. Consistently with previous extension proposals, the general principles, terminology, and data structure that define BIDS are shared amongst MRI, EEG, fNIRS and other modalities, thus enabling the reuse of existing software and tools, and providing support for multimodal imaging with little additional investment by researchers. As such, all data (i.e., monomodal or multimodal neuroimaging, and behavioral) from each subject are organized hierarchically in subject-specific subdirectories (e.g., *sub-01/, sub-02/, etc*.) (Fig. 1) and, if applicable, in session-specific subdirectories (e.g., *sub-01/ses-01/, sub-01/ses-02/*, etc.). Within each subject folder (or session folder, if present), there are modality-specific directories, such as *eeg* and *nirs* containing the actual data files.

Each NIRS-BIDS dataset must include a *dataset_description.json* file that provides a descriptive overview of the study, including information about authors and acknowledgments^7^. A tabular file *participants.tsv* must also be included to list all study participants alongside optional demographic information such as, for instance, their age, as to enable study-specific statistical modeling or to select age-specific optical pathlength factors^7,15^. Optionally, original non-BIDS datafiles (e.g., legacy or proprietary file formats, etc.) can be stored in the sub-directory *sourcedata*^12^, any conversion or processing code may be stored in the sub-directory *code* to facilitate replication^12^, and results of post-processing methods (i.e., tomographic image reconstruction) can be stored in the sub-directory *derivatives*^7^.

Both data and metadata for each measurement session are stored within the *nirs* subdirectory for each subject, and the files in this directory must use a specific naming pattern to encode relevant metadata (Fig. 1). For example, measurement data for subject YY (as an ordinal number) performing task XX would be stored as *sub-YY_task-XX_nirs.snirf*, with the file being compliant to SNIRF^6^. If available, session and run number will also be included in the filename as described in the general BIDS specification (e.g., *sub-YY_ses-1_run-1_task-XX_nirs.snirf*). Additionally, metadata files are stored alongside the measurement file to describe the acquisition parameters and montage information. Specific to NIRS-BIDS datasets, a *_*nirs.json* file (e.g., *sub-YY_task-XX_nirs.json*) describes the fNIRS data acquisition setup details and experimental task. A *_channels.tsv file specifies the pairing of optodes and channel properties such as wavelength and sampling frequency, amongst others (see Fig. 1). Recommended, yet non-mandatory files *_optodes.tsv and *_coordsystem.json describe the details of the optode type and the recorded and/or a standard coordinate system location for each optode, respectively (see Fig. 1). Optionally, if the experimental design included paradigm-specific events, these must be included in a *_events.tsv file that includes details about onset time, trial type, duration, and responses, amongst other details. Together, these files comprehensively describe the measurement data for subsequent analysis and sharing.

### Specific NIRS-BIDS considerations

The NIRS-BIDS proposal was developed to address the needs of the majority of the fNIRS community and the wide range of commercially available and research-grade custom-built fNIRS devices. Currently, the specification targets datasets acquired using continuous-wave fNIRS systems, which is the most widely deployed NIRS technique in research labs. However, the current NIRS-BIDS specification can further be extended as alternative fNIRS techniques (such as frequency- or time-domain systems) also become widely available.

Although a variety of vendor-specific file formats might still be in circulation for storing of NIRS data, only the SNIRF format (https://github.com/fNIRS/snirf) is supported by the NIRS-BIDS specification. SNIRF is a community-developed initiative supported by the Society for functional Near-Infrared Spectroscopy (https://fnirs.org, the worldwide professional society focused on NIRS) to create a single file format for the storage of all NIRS data and metadata^6^. It supports continuous wave, time- and frequency-domain NIRS data and was developed in collaboration between multiple software developers, device manufacturers, and academic institutions. The BIDS metadata files replicate some information included in the SNIRF file; this controlled redundancy is purposely included to provide independence from the SNIRF format and to enable easier human and machine reading of commonly queried metadata, thus improving findability^13^. Importantly, although the definition of SNIRF format predates the BIDS initiative and it technically allows storing multiple recordings in the same file, BIDS- compliant SNIRF files must contain data from a single run only.

A unique aspect of fNIRS is the concept of *source* and *detector* optodes. While other terms such as *transmitter* and *receiver* are commonly used by fNIRS researchers, the NIRS-BIDS specification uses the term *source* to refer to a light emitting device, *detector* to refer to a photoelectric transducer, and *optode* refers to either a source or detector. Since NIRS is a spectroscopic technique, its principle of operation depends on multiple frequencies or wavelengths of infrared light. Although fNIRS researchers commonly refer to a *channel* (or *optical channel*) as the pairing of a source and a detector regardless of the number of wavelengths being used, NIRS-BIDS defines a channel as an optical signal associated to a single wavelength of light for compatibility with the existing BIDS standard. Thus, a BIDS-formatted dataset typically contains two or more channels with different wavelengths associated to a single source-detector pair.

The NIRS-BIDS specification allows storage of optode positions either as measured locations, for example with an electromagnetic digitizer (x, y, and z coordinates) or as standardized coordinate locations (e.g., std_coord_x, std_coord_y, std_coord_z coordinates) referred, for instance, to the MNI atlas coordinates^16^. Anatomically-correct three-dimensional coordinates are preferred; however, due to historical reasons, many fNIRS datasets only contain two-dimensional information representing a flattened layout over a template head. In this situation, template x- and y-coordinates should be provided, and the template z-coordinate should be set to *n/a*. As with other neuroimaging modalities, a variety of coordinate systems supported by the BIDS specification can be used (https://bids-specification.readthedocs.io/en/stable/appendices/coordinate-systems.html#coordinate-systems), as well as custom coordinate systems, all to be described and utilized in the file *_coordsystem.json.

Another feature specific to fNIRS is the concept of *short separation channels* (equivalently, *short channels*), where closely spaced source-detector pairs are purported to measure extracerebral hemodynamic activity^17,18^. Due to the extensive variation in how manufacturers implement short channels (i.e., using dedicated sources, detectors, or headgear mechanisms), these can be described in the *_*nirs.json* file under the fieldname *ShortChannelCounts*. In addition, a column named *short_channel* is provided in the *_*channels.tsv* file for marking specific channels as *short*.

Several commercial vendors produce fNIRS devices with integrated auxiliary sensors such as accelerometers, gyroscopes, magnetometers, heart rate, and breathing-rate sensors, amongst others. If these additional data are acquired with the same integrated device, they should be stored alongside the fNIRS data in the same data file (i.e. in the aux field in the SNIRF data file). However, additional data simultaneously recorded with a different device should be stored according to their appropriate modality specification. For example, if heart rate data is measured with a separate device, it should be organized according to the physiological recordings specification in BIDS (https://bids-specification.readthedocs.io/en/stable/04-modality-specific-files/06-physiological-and-other-continuous-recordings.html), and not be saved in the SNIRF file.

### NIRS-BIDS data samples

We hereby describe four complete NIRS-BIDS example datasets that are publicly available and freely accessible to download from the Open Science Framework (OSF)^19^, Zenodo, and the Donders Repository (https://data.donders.ru.nl). Additionally, several example datasets with zero-byte data files are provided in the BIDS GitHub repository, which are useful for testing tools and services (https://github.com/bids-standard/bids-examples).

*Automatic and non-automatic finger and leg movements* (Cockx *et al*.^20^, 10.34973/vesb-mh30): A dataset including fNIRS data of 24 participants performing automatic and non-automatic finger and foot tapping movements in a block design. The task consisted of tapping four right-hand fingers on a keyboard or tapping the right foot on four squares on the floor in a specific order given by a 12-digit sequence (e.g., 434141243212). The participants executed two different sequences: a beforehand learned (i.e., automatic) version and a newly learned (i.e., non-automatic) version; under two conditions: with a dual-task (i.e., counting the letter ‘G’ from a list of letters appearing on the screen) or without a dual-task. fNIRS data was sampled with a multichannel fNIRS device (24 long and 12 short separation channels) over the left primary motor cortex, the left premotor cortex, the left and right dorsolateral prefrontal cortex, and the left and right posterior parietal cortex.

*Motor Task* (Luke and McAlpine, 10.5281/zenodo.5529797): Example dataset from five participants performing a finger-tapping experiment. Data were acquired with eight sources and eight detectors. An additional eight short-channel detectors were included in the montage. The optodes were placed over the motor cortex. The experiment was a block design with three conditions, (1) tapping the thumb to fingers on the left hand, (2) tapping the thumb to fingers on the right hand, and (3) no movement of thumb or fingers.

*Passive Auditory Task* (Luke *et al*.^21^, 10.17605/OSF.IO/F6TDK): Example dataset from seventeen participants performing a passive listening task. Data were acquired with sixteen sources and sixteen detectors, and the optodes were placed over the auditory cortices, occipital lobe, and left inferior frontal gyrus. An additional eight short-channel detectors were included in the montage. The experiment was a block design with three conditions (1) an acoustic speech stimulus, (2) an acoustic noise stimulus, (3) no auditory stimulus.

*Active Visual and Auditory Tasks* (Shader *et al*.^22^, 10.17605/OSF.IO/U23RB): Example data from an active speech comprehension task using both auditory- and visual-speech conditions. Data were acquired on 17 participants with sixteen sources and sixteen detectors. An additional eight short-channel detectors were included in the montage. The optodes were placed over the auditory cortices, occipital lobe, and left inferior frontal gyrus. The experiment was a block design with three conditions (1) speech presented acoustically, (2) speech presented visually, and (3) no auditory or visual stimuli.

### NIRS-BIDS Tools

The *bids-validator* tool can be used to validate that a newly formatted dataset adheres to the BIDS specification, such as checking for missing data or underspecified metadata. The validator (https://bids-standard.github.io/bids-validator/) works directly in a web-browser without requiring the upload of any data files.

A dedicated tool has been developed to simplify the conversion of fNIRS data to BIDS format (Luke and McAlpine, 2021a). Additionally, the BfNIRS Cloud, an active online platform, has been developed to facilitate the creation, editing, validation, and sharing of BIDS-compliant fNIRS datasets (https://www.bfnirs.openfnirs.org/). The majority of the larger fNIRS software processing platforms provide support for reading fNIRS data in the BIDS format. The ability to read NIRS-BIDS formatted datasets is provided by MATLAB packages Homer3^23^, FieldTrip^24^, Brainstorm^25^, QT-NIRS^26^, Brain AnalyzIR^27^ and in Python using MNE-Python ecosystem with MNE-BIDS and MNE-NIRS^21,28–30^. Together with the NIRS-BIDS standard, these tools enable researchers to utilize the strengths of each software package for quality assurance and analysis. For example, a researcher could collect and save data in BIDS format, then examine the data quality using the dedicated QT-NIRS tool, then conduct pre-processing in FieldTrip, and apply a GLM analysis using MNE-NIRS.

## Discussion

The development of NIRS-BIDS is arguably a critical advancement towards a wider dissemination of fNIRS datasets that, in comparison to other neuroimaging modalities, is still fairly limited. Amongst other reasons for such a limited diffusion is the fact that functional NIRS is inherently prone to a wider variety of non-standardized technical and experimental features such as optode layouts and their pairings, NIRS techniques (CW, TD or FD), and instrument designs, resulting in datasets that are not easily comparable and reusable by other researchers. Both the SNIRF file format and the NIRS-BIDS data structure precisely address these issues by describing such a variety of metadata in a well-organized, readable and parsable fashion that will unequivocally facilitate data sharing. Notably, both these efforts have been proposed and officially approved only recently (SNIRF in 2022, and NIRS-BIDS in 2023), hence adoption is expected to spread widely in the near future. The Society for fNIRS committees and several regional NIRS groups regularly advocate for standardization in their conferences and workshops, and all leading device manufacturers currently allow saving fNIRS scans in SNIRF format. In addition, the ever-growing number of multimodal studies integrating fNIRS and EEG will naturally be facilitated by the ability to organize datasets from both techniques in a single, straightforward hierarchy, which will result in further adoption of NIRS-BIDS.

As the field of continuous-wave NIRS evolves towards high-density layouts with featuring dozens of optodes, hundreds or thousands of optical channels, and increasingly common use of short separation channels, the metadata organization offered by NIRS-BIDS will have a significant impact on the development of new analytical tools, data sharing practices, and the advancement of open science. A wider dissemination of NIRS-BIDS datasets will also enable additional research aimed at improving reliability and validity of fNIRS datasets at a scale achieved by other neuroimaging modalities^31,32^. Moreover, the promising extension of NIRS-BIDS to include time-domain and frequency-domain NIRS represents a significant advancement, especially given that many devices already support data and metadata storage in the SNIRF format.

## Methods

Commonly to many other BEP efforts, NIRS-BIDS started with a virtual meeting in which the objective and process of BIDS was described to a group of NIRS investigators and vendors part of the Society of fNIRS collaborative network. Following the initial enthusiastic support of extending the BIDS standard to NIRS datasets, a smaller group of participants started interacting with the BIDS governance and developing the formal BEP proposal, to which the community at large was periodically invited to contribute additions and comments. This process was partly facilitated and guided by the existing development effort of the SNIRF format that at that time was nearly complete, but also partly challenged by the conflict or redundancy of content between the SNIRF format and common principles of BIDS, and by the diversity of approach and terminology between NIRS and other approved BEP extensions, primarily EEG and MEG. Critically, the active participation of developers of other BEPs and of BIDS maintainers, acknowledged as authors of this manuscript, was critical to the resolution of all issues.

Upon reaching a final consensus, the BEP proposal was submitted for review by BIDS Steering Group, concurrently to the preparation and publication of NIRS-BIDS public examples and the update of the BIDS validator. Lastly, NIRS-BIDS was merged into the BIDS standard and released as version 1.8.0, and fully described at https://bids-specification.readthedocs.io/en/stable/modality-specific-files/near-infrared-spectroscopy.html

To facilitate the adoption of NIRS-BIDS by the readership, we hereby summarize the main steps needed to create a BIDS dataset starting from raw NIRS data.

### Creating a BIDS-compliant dataset

In a fashion analogous to the original BIDS description^7^, the process of creating a NIRS-BIDS compatible dataset can be achieved as follows:

- *Step 1: Convert proprietary NIRS files to.snirf*. Before the development of the SNIRF format, most vendors defined their own data file formats, with several of these still existing to date whilst adoption of standards takes over. However, NIRS-BIDS acknowledges.snirf as the only standard NIRS file format, hence conversion from any proprietary format to SNIRF is mandatory. Fortunately, several commercial and academic software tools can read original formats and rewrite data as.snirf standard.
- *Step 2: Create folder structure, rename and copy snirf files*. This implies creating and/or reorganizing and (re-)naming files to ensure compliance with the NIRS-BIDS folder structure and naming scheme. We are aware of several ad-hoc scripts already being used by some early-adopter groups of our community to automatize this process and it is expectable that they will be incorporated in the most prominent software tools very soon.
- *Step 3: Add auxiliary or multimodal data (as applicable)*. Although the SNIRF format has some capacity to accommodate auxiliary data and/or experimental stimuli description, actual experimental data may exceed that capacity. For instance, multimodal EEG-NIRS datasets or audio/videos stimulation cannot be fully included in a.snirf file and it is not advisable to do so, especially when BIDS can accommodate additional data in a standardized fashion. Hence, NIRS-BIDS requires that additional data to be provided in form of complementary modality-specific BIDS directories (e.g., ‘eeg’) or in terms of additional stimuli information and files such as.wav,.mov, etc. (https://bids-specification.readthedocs.io/en/stable/modality-specific-files/task-events.html#stimuli)
- *Step 4: Add any missing metadata*. This refers to experiment-level (as opposed to file level) metadata. The original BIDS standard mentions, as examples, ontological links (e.g., the NIRS Glossary Project at https://fnirsglossaryproject.github.io/ or OntoNIRS at https://osf.io/dsjpz/), atlas information, the list of authors, or ways to reference the dataset, among others.
- *Step 5: Validate the dataset*. The last step is to validate the dataset using the BIDS validator (see link in the Code Availability section), which will check whether required files or required data are missing, as well as data type and value compliance.

## Data Availability

Datasets conforming with NIRS-BIDS are available at: 10.34973/vesb-mh30^33^, CC-BY-SA 10.5281/zenodo.5529797^34^, CC-BY-SA 10.17605/OSF.IO/F6TDK^35^, CC-BY-SA 10.17605/OSF.IO/U23RB^36^, CC-BY-SA

## Code availability

The BIDS validator code is available in the BIDS Validator repository on GitHub at https://github.com/bids-standard/bids-validator.

## References

[1] Boas, D. A., Elwell, C. E., Ferrari, M. & Taga, G. Twenty years of functional near-infrared spectroscopy: introduction for the special issue. *NeuroImage* **85**, 1–5 (2014). 24321364 10.1016/j.neuroimage.2013.11.033

[2] Ferrari, M. & Quaresima, V. Review: Near infrared brain and muscle oximetry: From the discovery to current applications. *Journal of Near Infrared Spectroscopy* **20**, 1–14 (2012).

[3] Scholkmann, F. *et al*. A review on continuous wave functional near-infrared spectroscopy and imaging instrumentation and methodology. *NeuroImage* **85 Pt 1**, 6–27 (2014). 23684868 10.1016/j.neuroimage.2013.05.004

[4] Ayaz, H. *et al*. Optical imaging and spectroscopy for the study of the human brain: status report. *NPh* **9**, S24001 (2022). 10.1117/1.NPh.9.S2.S24001 PMC9424749 36052058

[5] Yücel, M. A., Selb, J. J., Huppert, T. J., Franceschini, M. A. & Boas, D. A. Functional Near Infrared Spectroscopy: Enabling Routine Functional Brain Imaging. *Curr Opin Biomed Eng* **4**, 78–86 (2017). 29457144 10.1016/j.cobme.2017.09.011 PMC5810962

[6] Tucker, S. *et al*. Introduction to the shared near infrared spectroscopy format. *Neurophotonics* **10**, 013507 (2023). 36507152 10.1117/1.NPh.10.1.013507 PMC9732807

[7] Gorgolewski, K. J. *et al*. The brain imaging data structure, a format for organizing and describing outputs of neuroimaging experiments. *Sci Data* **3**, 160044–160044 (2016). 27326542 10.1038/sdata.2016.44 PMC4978148

[8] Holdgraf, C. *et al*. iEEG-BIDS, extending the Brain Imaging Data Structure specification to human intracranial electrophysiology. *Sci Data* **6**, 102–102 (2019). 31239438 10.1038/s41597-019-0105-7 PMC6592874

[9] Knudsen, G. M. *et al*. Guidelines for the content and format of PET brain data in publications and archives: A consensus paper. *J Cereb Blood Flow Metab* **40**, 1576–1585 (2020). 32065076 10.1177/0271678X20905433 PMC7370374

[10] Moreau, C. A. *et al*. The genetics-BIDS extension: Easing the search for genetic data associated with human brain imaging. *Gigascience* **9**, giaa104 (2020). 33068112 10.1093/gigascience/giaa104 PMC7568436

[11] Niso, G. *et al*. MEG-BIDS, the brain imaging data structure extended to magnetoencephalography. *Sci Data* **5**, 180110–180110 (2018). 29917016 10.1038/sdata.2018.110 PMC6007085

[12] Pernet, C. R. *et al*. EEG-BIDS, an extension to the brain imaging data structure for electroencephalography. *Sci Data* **6**, 103–103 (2019). 31239435 10.1038/s41597-019-0104-8 PMC6592877

[13] Wilkinson, M. D. *et al*. The FAIR Guiding Principles for scientific data management and stewardship. *Sci Data* **3**, 160018–160018 (2016). 26978244 10.1038/sdata.2016.18 PMC4792175

[14] Zuo, X.-N., Xu, T. & Milham, M. P. Harnessing reliability for neuroscience research. *Nature Human Behaviour* **3**, 768–771 (2019). 31253883 10.1038/s41562-019-0655-x

[15] Scholkmann, F. & Wolf, M. General equation for the differential pathlength factor of the frontal human head depending on wavelength and age. *Journal of Biomedical Optics* **18**, 105004 (2013). 24121731 10.1117/1.JBO.18.10.105004

[16] Okamoto, M. *et al*. Three-dimensional probabilistic anatomical cranio-cerebral correlation via the international 10–20 system oriented for transcranial functional brain mapping. *NeuroImage* **21**, 99–111 (2004). 14741647 10.1016/j.neuroimage.2003.08.026

[17] Saager, R. B. & Berger, A. J. Direct characterization and removal of interfering absorption trends in two-layer turbid media. *Journal of the Optical Society of America A* **22**, 1874 (2005). 10.1364/josaa.22.001874 16211814

[18] Tachtsidis, I. & Scholkmann, F. False positives and false negatives in functional near-infrared spectroscopy: issues, challenges, and the way forward. *Neurophotonics* **3**, 031405–031405 (2016). 27054143 10.1117/1.NPh.3.3.031405 PMC4791590

[19] Foster, E. D. & Deardorff, A. Open Science Framework (OSF). *Journal of the Medical Library Association* **105** (2017).

[20] Cockx, H. *et al*. fNIRS is sensitive to leg activity in the primary motor cortex after systemic artifact correction. *NeuroImage* 119880 10.1016/j.neuroimage.2023.119880 (2023). 10.1016/j.neuroimage.2023.119880 36693595

[21] Luke, R. *et al*. Analysis methods for measuring passive auditory fNIRS responses generated by a block-design paradigm. *Neurophotonics* **8**, 025008–025008 (2021). 34036117 10.1117/1.NPh.8.2.025008 PMC8140612

[22] Shader, M. J., Luke, R., Gouailhardou, N. & McKay, C. M. The use of broad vs restricted regions of interest in functional near-infrared spectroscopy for measuring cortical activation to auditory-only and visual-only speech. *Hearing Research* **406**, 108256 (2021). 34051607 10.1016/j.heares.2021.108256

[23] Huppert, T. J., Diamond, S. G., Franceschini, M. A. & Boas, D. A. HomER: a review of time-series analysis methods for near-infrared spectroscopy of the brain. *Applied optics* **48**, 280–298 (2009). 10.1364/ao.48.00d280 PMC2761652 19340120

[24] Oostenveld, R., Fries, P., Maris, E. & Schoffelen, J.-M. FieldTrip: Open source software for advanced analysis of MEG, EEG, and invasive electrophysiological data. *Comput Intell Neurosci* **2011**, 156869–156869 (2011). 21253357 10.1155/2011/156869 PMC3021840

[25] Tadel, F., Baillet, S., Mosher, J. C., Pantazis, D. & Leahy, R. M. Brainstorm: a user-friendly application for MEG/EEG analysis. *Comput Intell Neurosci* **2011**, 879716–879716 (2011). 21584256 10.1155/2011/879716 PMC3090754

[26] Hernandez, S. M. & Pollonini, L. NIRSplot: A Tool for Quality Assessment of fNIRS Scans. 10.1364/brain.2020.bm2c.5 (Optica Publishing Group, 2020).

[27] Santosa, H., Zhai, X., Fishburn, F. & Huppert, T. The NIRS Brain AnalyzIR Toolbox. *Algorithms* **11**, 73 (2018). 38957522 10.3390/a11050073 PMC11218834

[28] Appelhoff, S. *et al*. MNE-BIDS: Organizing electrophysiological data into the BIDS format and facilitating their analysis. *J Open Source Softw* **4**, 1896 (2019). 35990374 10.21105/joss.01896 PMC9390980

[29] Gramfort, A. *et al*. MEG and EEG data analysis with MNE-Python. *Front Neurosci* **7**, 267–267 (2013). 24431986 10.3389/fnins.2013.00267 PMC3872725

[30] Gramfort, A. *et al*. MNE software for processing MEG and EEG data. *Neuroimage* **86**, 446–460 (2014). 24161808 10.1016/j.neuroimage.2013.10.027 PMC3930851

[31] Zuo, X.-N. & Xing, X.-X. Test-retest reliabilities of resting-state FMRI measurements in human brain functional connectomics: A systems neuroscience perspective. *Neuroscience & Biobehavioral Reviews* **45**, 100–118 (2014). 24875392 10.1016/j.neubiorev.2014.05.009

[32] Nichols, T. E. *et al*. Best practices in data analysis and sharing in neuroimaging using MRI. *Nature Neuroscience* **20**, 299–303 (2017). 28230846 10.1038/nn.4500 PMC5685169

[33] Cockx, H. *et al*. Automatic and non-automatic finger and leg movements measured with functional near-infrared spectroscopy (fNIRS). Radboud University 10.34973/vesb-mh30 (2023).

[34] Luke, R. & McAlpine, D. fNIRS Finger Tapping Data in BIDS Format. *Zenodo*10.5281/zenodo.6575155 (2022).

[35] Luke, R., Shader, M., Larson, E. & Innes-Brown, H. Analysis methods for measuring passive auditory fNIRS responses generated by a block-design paradigm. *OSF*10.17605/OSF.IO/F6TDK (2021). 10.1117/1.NPh.8.2.025008 PMC8140612 34036117

[36] Luke, R. & Shader, M. fNIRS Audio & Visual Speech. Broad vs Restricted Regions of Interest. *OSF*10.17605/OSF.IO/U23RB (2021).

